# Naval Medicine in the Great War
*A Paper read before the Medical Society of London on February 12, under the presidency of Lieutenant-Colonel D'Arcy Power, F.R.C.S., R.A.M.C.(T.).


**Published:** 1917-02-17

**Authors:** H. D. Rolleston


### NAVAL MEDICINE IN THE GREAT WAR.*

By SURGEON-GENERAL H. D. ROLLESTON, C.B., M.D. (Cantab.), F.R.C.P.

At the outbreak of the war, the permanent Medical Service of the Navy, under the Medical Directorship of Sir Arthur May, was at once expanded by the mobilisation of its retired members, of officers on the emergency list, and of the medical officers on the Royal Naval Volunteer Reserve. Many temporary surgeons were also entered, and some ten consultants were appointed, mainly to the large base hospitals. A considerable proportion of temporary surgeons were passed through the large naval hospitals, where they received instruction in naval routine. At a later stage, unqualified medical students who had passed in anatomy and physiology were appointed under the title "Surgeon-Probationer R.N.V.R." Their instruction at Haslar included Service routine, victualling of the sick, and the treatment of wounded in action. These men were subsequently drafted to destroyers and other small sea-going ships, to render first-aid. After a certain period of service, many of them were granted leave to prepare for and pass their final medical examination, which entitled them to rank as temporary surgeons. The clinical laboratories at large base hospitals were partly staffed by men with laboratory knowledge, but not possessing medical qualifications. These did not receive any naval rank. Dr. P. Fildes, who was sent to Haslar by the Medical Research Committee, has been of the greatest service in dealing with bacteriological work.

Queen Alexandra's Royal Naval Nursing Service was increased to three times its former strength by the mobilisation of the Reserve nursing sisters. The existing berth staff, which had been much depleted when the Fleet was mobilised, was supplemented by the Naval Auxiliary Sick-berth Reserve, composed mostly of men from the St. John Ambulance Brigade.

### The Transport of Wounded and Sick.

The Naval Transport had been under the control of Surgeon-General Sir James Porter, K.C.B., who, early in the year 1915, went out to supervise the transport arrangements in Gallipoli, both for the Navy and Army. A number of new methods of transport has been employed, among them being Boydon's new ambulance sling, a mono-wheel stretcher-carrier (which was the invention of the Rev. B. Close), the employment of the movable-cot system on ambulance trains, and several improvements on hospital ships.

The ambulance trains, which travel as required from North to South, carry two medical officers, and a crew of thirty-six stewards and sick-berth attendants, but no female nurses. A small ambulance train, consisting of six coaches, travels locally between the naval bases and the local hospitals.

With regard to hospital ships in modern naval engagements, the hospital ships cannot accompany the Fleet and take charge of the wounded. There are several reasons for this. First, the hospital ships would be unable to keep pace with the rapidly-moving battleships. Secondly, it would only be possible to transfer wounded men from a battleship to a hospital ship in exceptionally calm weather. Thirdly, such transfer would require that the battleship should heave to, which would expose it to hostile submarine attack. To meet this need it has been suggested (though, so far, it has not been carried out) that fast vessels, of about 500 tons, registered under the Geneva Convention and fitted with appliances for rapidly picking up survivors from the sea, should be employed on the scene of a recent action.

During a hospital ship's stay at a naval base, a large number of surgical operations are carried out on it, and minor cases, when cured, return thence direct to the Fleet. After an action, the hospital ships are collected at the naval base to await the arrival of the battleships, receive the wounded and serve as clearing-stations, and evacuate cases to the shore. Severe cases, which would suffer from transport, are taken to the smaller hospitals in the immediate neighbourhood. Hence, immediately after a battle, the hospital ship ceases to act as a base hospital, and becomes a clearing-station.

A hospital ship may be employed as a permanent floating hospital for infectious diseases at a naval base which is remote from such hospitals on land.

### The Health of the Navy.

Dr. Rolleston arranged his treatment of this important question under three heads: (1) Shore Depôts and Training Establishments; (2) The Service Afloat; (3) Royal Naval Division and the Marines Serving on Land.

With regard to shore depôts and training establishments, these and the barracks are more exposed than the Service afloat to epidemic and other infections, on account of their contact with the civilian population, especially owing to the massing together of large numbers of youths, who are more susceptible than are adults to the common epidemic diseases. The health of the Fleet has been protected by quarantine measures concerning drafts sent to it from depôts. The liability to sickness of the shore establishments is shown by the incidence of cerebro-spinal fever, for of the 272 cases during the war, 252 (92 per cent.) occurred on shore.

In reference to the Service afloat, the general health of the Grand Fleet has been extremely good—probably better than in times of peace. In 1913, the average daily percentage of sick in the whole Fleet was 2.37; in 1914, 2.03. The average daily sickness in the Grand Fleet has always been under 1 per cent., and most of the sickness has been of a minor character. Both in the Fleet and among Reservists there has been an epidemic of measles, but there has been no case of small-pox. Except

* A Paper read before the Medical Society of London on February 12, under the presidency of Lieutenant-Colonel D'Arcy Power, F.R.C.S., R.A.M.C.(T.).

in the Mediterranean Fleet, there have been very few cases of enteric fever.

In the Eastern Mediterranean Squadron, from August to November, 1915, nearly every ship's crew was attacked by epidemic gastro-enteritis. The disease died down in December. The infection was probably conveyed by flies and by men returning from the beach.

The health of the Adriatic Squadron at this time was excellent. The average daily sickness for the whole Mediterranean Fleet from August 15 to October 31, 1915, was 2.24 per cent.; while from November 1, 1915, to January 31, 1916, it had improved to 1.52 per cent. Sand-fly fever was prevalent at Salonika in May.

The good bill of health of the Fleet as a whole, Surgeon-General Rolleston declared to be due to the following factors: (1) the comparative isolation of the Fleets, especially the Grand Fleet, enabling the crews to be free of the dangers appertaining to venereal disease and alcohol at ports. (2) Quarantine precautions in drafting from shore establishments to the Fleet, which were instituted by Sir Arthur May. Lectures to the ships' crews on personal hygiene, dealing specially with the dangers of sex disease and alcoholic excess. That these have had a good effect is testified by the fact that in 1,100 men returning from leave there were only three cases of gonorrhœa, and one case of syphilis. (4) Measures, such as entertainments and sports, to counteract the effect of the monotony.

In a number of cases the hard physical requirements led, in young subjects, to the activation of latent disease, such as tuberculosis and heart affections. The present war seems to have shown an increase in cases of exophthalmic goitre. A similar increase was noted in the siege of Paris in 1870 and in the Boer War.

To the prolonged and monotonous strain of naval work must also be attributed a number of cases of psychasthenia and neurasthenia, especially in those of neurotic taint who have undergone long training. In the senior officer the burden of responsibility, combined with the comparative isolation, may lead to a want of confidence, and may breed brain-fag. Yet there have been less than 1 per cent. of serious cases of mental trouble, and only 4 per cent. of cases of mild neurasthenia. There was a marked absence of this among the men after the Jutland battle.

### Casualties during Action.

In sea battles, in comparison with land warfare, a striking number of the men hit are killed outright. This was so in the Russo-Japanese War. There is no soil-contamination of wounds, as in land battles; the wounds seen in a naval battle are comparable to severe machinery wounds seen in workshops. At least one-third of the casualties are burns, and these fall mostly into two categories: in the first are burns due to the ignition of our own cordite, or to burning furniture. Long-continued exposure to these flames results in a deep destruction of tissues, and the immediate mortality in these cases is high. The second category embrace burns to the exposed skin of face and hands due to the momentary flash of high-explosive shells in confined spaces. Shock, even in slight cases, was found to be excessive, and for this reason immediate operations should be avoided. Morphine, half to two-thirds of a grain hypodermically, was the sheet-anchor in avoiding shock.

In the poisoning by gases liberated from explosives the most powerful factor is nitrous oxide, and the symptoms of this come on after a comparatively uneventful interval. The prophylaxis employed is quite effective—namely, a Service respirator containing cotton-waste soaked in the usual soda solution. When once established, however, the treatment of the condition is unsatisfactory There have also been instances of carbon monoxide poisoning. Appendicitis is not rare among the *personnel*, and it showed an increase in the ships which took part in the Jutland battle.

In the Gallipoli campaign, diarrhœa was so frequent that it was regarded as an incident rather than as a cause of declaring sick. Dysentery claimed many victims, but treatment by emetine and anti-dysenteric serum gave very satisfactory results. A form of peripheral neuritis identical with that of beri-beri in its clinical aspects occurred in Gallipoli and in Mesopotamia. The Royal Naval Division enjoyed freedom from cholera, due to the anti-choleraic inoculation; this disease was prevalent among the Turks in the neighbourhood. With regard to diseases among airmen, dizziness may be produced by the leakage of petrol spray, while the exhaust gases from the engine may cause headache, drowsiness, and malaise. One airman, who had been exposed to 34° of frost at 15,000 feet, suffered from frost-bite. Occasionally the development of psychasthenic symptoms shows that the airman has mistaken his vocation.

### After-Comments.

Sir Arthur May (Medical Director-General of the Navy) spoke of the great interest with which he had listened to Surgeon-General Rolleston's Paper. He could scarcely be expected to criticise it, and he did not propose to eulogise it. They had no business to boast themselves until the time came for putting off their armour. The Naval Medical Service was much indebted to the medical men from civilian practice who had joined it since the war began, for the health of the Navy had been maintained at a remarkably high level. Only that day he noticed that in the report on 20,000 men the percentage of disease was just under 0.6, as against a peace-time percentage of 2. This meant that instead of there being 400 sick in 20,000 there were only 120—a great result, remembering the value of men at the present time. This result he considered to be due to the infinite and unremitting care taken by the medical officers in charge of the crews. Those gentlemen of the profession who joined the Service from outside on the outbreak of war had taken to the special conditions met with in the Navy with wonderful celerity; and his high praise must also be taken to apply to the surgeon-probationers.

